# Relationship between areal BMD, FRAX®, and femoral strength in community-dwelling older Asian adults

**DOI:** 10.1007/s11657-025-01617-1

**Published:** 2025-11-04

**Authors:** Dheeraj Jha, Manju Chandran, Dario Koller, Vee San Cheong, Anitha D. Praveen, Alexander Baker, Preeti Gupta, Ecosse L. Lamoureux, Halldór Pálsson, Stephen J. Ferguson, Benedikt Helgason

**Affiliations:** 1 Future Health Technologies Programme, Singapore-ETH Centre, CREATE Campus, 1 Create Way, CREATE Tower, #06-01, Singapore, 138602 Singapore; 2 Institute for Biomechanics, ETH-Zürich, Zürich, Switzerland; 3 Duke-NUS Medical School, Singapore, Singapore; 4 Osteoporosis and Bone Metabolism Unit, Department of Endocrinology, Singapore General Hospital, Singapore, Singapore; 5 Insigneo Institute, University of Sheffield, Sheffield, UK; 6 School of Mechanical, Aerospace and Civil Engineering, University of Sheffield, Sheffield, UK; 7 Singapore Eye Research Institute (SERI), Singapore, Singapore; 8 University of Iceland, Reykjavik, Iceland

**Keywords:** Hip fracture, Dual-energy X-ray absorptiometry, FRAX, Femoral strength, Finite element modeling

## Abstract

**Summary:**

*T*-scores alone are inadequate for identifying hip fracture risk. Incorporating FRAX-HFP scores and femoral strength improves risk assessment. Tailored interventions are needed for different ethnicities, with a focus on females due to higher fracture risk. Sex-specific thresholds and targeted prevention strategies are essential for effective fracture prevention.

**Background:**

We investigated the age-related trajectories of areal bone mineral density (aBMD), fracture risk assessment tool (FRAX)–based 10-year probability of hip fracture (FRAX-HFP), trochanteric soft tissue thickness (TSTT), and femoral strength in a multi-ethnic cohort of community-dwelling older adults in Singapore. We also examined the relationship between FRAX-HFP and femoral strength.

**Methods:**

Dual-energy X-ray absorptiometry (DXA) scans were conducted for Singaporean older adults (*n* = 2235), enrolled in the Population Health and Eye Disease Profile in Elderly Singaporeans (PIONEER) study. aBMD and FRAX-HFP were recorded for the subjects. TSTT was derived from whole-body DXA scans. Femoral strength was derived from DXA-based 3D finite element models. Age-related trajectories were compared for three major ethnicities in Singapore. The relationship between FRAX-HFP and femoral strength was examined.

**Results:**

The study included 2204 older adults (1224 females (73.71 ± 8.37 years), 980 males (73.45 ± 8.34 years)). Age-related trajectories for aBMD, FRAX-HFP, TSTT, and femoral strength indicated that Chinese ethnicity is at high risk for fracture, compared to Indians and Malays. Separately, FRAX-HFP identified 16% of males and 27% of females, and femoral strength identified 3% of males and 1% of females at risk. Both FRAX-HFP score and femoral strength identified 24% of males and 35% of females at risk.

**Conclusion:**

Age-related trajectories for aBMD, FRAX-HFP, TSTT, and femoral strength were found to be consistent with the hip fracture trends in Singapore. FRAX-HFP and femoral strength identified different individuals at risk, indicating that each, either alone or combined with aBMD, could improve the ability to assess hip fracture risk.

## Introduction

Osteoporosis is a chronic condition leading to a weakened bone structure due to loss of bone mass and deterioration in trabecular structural arrangement. This results in greater bone fragility and a higher risk of hip fractures with age [1]. Following a hip fracture, patients experience a 3-month long decline in physical, mental, and emotional function leading to disability in about 40% of cases [2]. When compared to an age-matched control group, the annual mortality rate of hip fracture patients was higher by 8% for females and 18% for males [3]. The current clinical standard for the diagnosis of osteoporosis is to use areal bone mineral density (aBMD) obtained from dual-energy X-ray absorptiometry (DXA) scans. The DXA scan-based *T*-score defines how much bone mass differs from the bone mass of an average healthy 25–30-year-old adult. However, 28–61% of hip incident fractures have been reported to occur in individuals with an aBMD measurement higher than the threshold defined for osteoporosis, which is a *T*-score of − 2.5 [4–6]. Thus, stratifying fracture risk based on aBMD alone lacks sensitivity for clearly identifying individuals at risk. The Fracture Risk Assessment Tool (FRAX) (https://frax.shef.ac.uk/FRAX/) is a calculator used for predicting the 10-year probability of a hip fracture or other major osteoporotic fracture considering 12 risk factors including aBMD. FRAX scores are tailored to specific countries and, in some instances, ethnic groups. For example, in China (including Hong Kong), Malaysia (with distinctions for Chinese, Bhumiputera, and Indian), Singapore (Chinese, Malay, Indian), South Africa (African, Colored, Indian, White), and the USA (Caucasian, Black, Hispanic, Asian), ethnic variations are considered. However, some evidence suggests that FRAX may not perform as well in Asian populations, compared to Caucasian populations [7, 8].

In addition to bone mass, bone shape, size, and bone material properties contribute to resistance to fracture [9]. Femoral strength predicted from image-based subject-specific finite element models (FEMs) has been studied in the past to evaluate bone’s resistance to fracture [10–17]. The early studies in this field seemed to suggest no improvement in using femoral strength for stratifying hip fracture risk compared to aBMD, but more recent research has shown FEMs to be more effective than aBMD [11, 16, 17], even demonstrating consistent superiority over 16 years of follow-up [17]. Prior studies were limited by small sample sizes, study designs, and short follow-up durations. However, advances in computational efficiency, automation, and modeling technologies now make large-scale analysis of femoral strength possible. Despite this, CT scans are associated with high radiation and cost and are not systematically used for primary screening for osteoporosis [18]. In parallel, 2D-3D registration techniques to build 3D FEMs of the proximal femur from lower radiation dose DXA scan have been developed [19, 20]. Two studies on Caucasian participants found that bone strength estimated from DXA-based finite element models (FEMs) had superior ability to distinguish individuals with hip fractures compared to aBMD [21, 22]. However, further validation with a larger, more diverse sample, including individuals from various ethnicities, is needed. The 2D-3D registration process is now available in a commercial software (3D-SHAPER®, Galgo Medical), which is increasingly being utilized in clinical practice [23]. Additionally, studies have been published where the software’s output has been employed to develop FEMs [24, 25].

In Asia, the number of hip fractures is estimated to rise from 1.12 to 2.56 million between 2018 and 2050, leading to an increase in the annual direct cost of hip fracture from USD 9.5 to 15 billion [26]. By the year 2050, more than half of the hip fractures are expected to occur in Asia [26]. Interestingly, Singapore has a high incidence rate of hip fractures, similar to that seen in Nordic countries in Europe. The standardized incidence rates per 100,000 population for individuals over 50 years old are 314.2 in Singapore, 189.5 in South Korea, 315.9 in Denmark, 226.5 in Finland, and 134 in the UK [27]. In our previous study involving 275 older adults, we found that trochanteric soft tissue thickness (TSTT) could partially explain the differences in fracture rates among various ethnic groups in Singapore [Jha et. al, Clin. Biomech. Under review]. How differences in femoral strength could inform on variations in hip fracture risk in older adults in Singapore across different ethnic groups is not known. Whether femoral strength, TSTT, FRAX-HFP, and aBMD identify the same or different individuals at risk for hip fractures is also not clear. Therefore, the primary aim of this study is to investigate the age-related trajectories (patterns or trends over time as people age) of aBMD, FRAX-HFP, TSTT, and femoral strength in a diverse, multi-ethnic cohort of community-dwelling older adults in Singapore. A secondary aim is to examine the relationships that femoral strength and FRAX-HFP have with *T*-scores to provide insight into whether the stratification using these biomarkers overlaps or whether a potential synergistic effect exists between them.

## Materials and methods

### Study cohort

The study involved 2643 older adults from the Population Health and Eye Disease Profile in Elderly Singaporeans (PIONEER) cohort [28], which includes community-dwelling Singaporeans aged 60 and above. Data collection took place from 2017 to 2022; the study was approved by the SingHealth Centralized Institutional Review Board (2016/3089). Subjects were included if their DXA scans and FRAX-HFP scores were available, resulting in a final sample of 2235 subjects. Demographic information collected included age, gender, height, weight, and ethnicity.

### aBMD measurements

Subjects underwent DXA scans, using the Hologic® Horizon W scanner (Hologic, Inc., Marlborough, MA, USA), of the proximal femur and whole body while lying in a supine position with their legs rotated inward at a 25° angle, as recommended by the manufacturer. This positioning is required to bring the femoral neck axis parallel to the plane of the scan table. Each scan was visually inspected for artifacts. The aBMD at the total hip and the *T*-score were retrieved from the scanning reports provided by the scanner software.

### FRAX-HFP calculations

FRAX-HFP scores, reflecting predicted 10-year probability of a hip fracture, were calculated based on 12 clinical risk factors for each subject (https://frax.shef.ac.uk/FRAX/). The clinical risk factors include age, gender, weight, height, history of fractures, having a parent with a hip fracture, current smoking, glucocorticoid use, rheumatoid arthritis, secondary osteoporosis, alcohol consumption, and aBMD.

### TSTT calculations

Standing trochanteric soft tissue thickness (TSTT) from supine whole-body DXA images was estimated using a prediction equation developed in a previous study [Jha et. al, Clin. Biomech. Under review]. Supine TSTT (TSTT_SUP_) measures are used as input in the gender and ethnic (Malay, Indian, and Chinese) specific equations (Eq. 1). These TSTT_SUP_ values were derived using a Python (v3.9) pipeline that processes the subject’s whole-body DXA image, utilizing the open-source Python package *OpenCV* (v4.6.0.66).

1 $${\varvec{T}}{\varvec{S}}{\varvec{T}}{\varvec{T}} = {\varvec{S}}{\varvec{l}}{\varvec{o}}{\varvec{p}}{\varvec{e}} *{{\varvec{T}}{\varvec{S}}{\varvec{T}}{\varvec{T}}}_{{\varvec{S}}{\varvec{U}}{\varvec{P}}}+{\varvec{I}}{\varvec{n}}{\varvec{t}}{\varvec{e}}{\varvec{r}}{\varvec{c}}{\varvec{e}}{\varvec{p}}{\varvec{t}}$$

TSTT : average trochanteric soft tissue thickness in standing position, cm
TSTT_SUP_: average trochanteric soft tissue thickness in supine position, cm
Slope: Chinese (males, 0.63; females, 0.59), Indian (males, 0.54; females, 0.51), Malay (males, 0.53; females, 0.62)
Intercept: Chinese (males, 3.12 cm; females, 2.29 cm), Indian (males, 10.54 cm; females, 12.26 cm), Malay (males, 8.94 cm; females, 6.23 cm)

### Femoral strength calculations

**Fig. 1:**
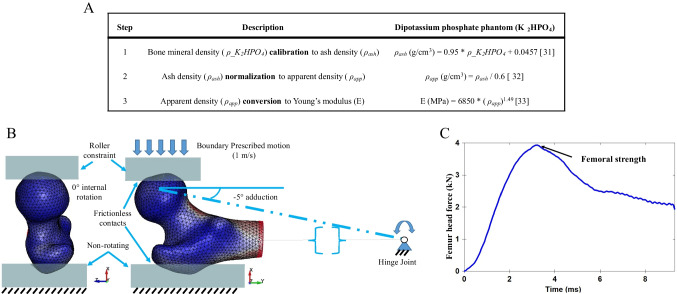
**A** Material mapping steps for the finite element models of the proximal femur. *ρ_K*_*2*_*HPO*_*4*_, bone mineral density in g/cm^3^; *ρ*_*ash*_, ash density; *ρ*_*app*_, apparent density; *E*, Young’s modulus. **B** Sample finite element model from the PIONEER cohort with boundary conditions for sideways fall loading. **C** Sample femur head force–time response recorded to measure femoral strength at the peak

The 3D volume and outer surface of the proximal femur from DXA scans were generated using 3D-SHAPER® (v2.11.1) software from the 2D DXA image [23]. The volume and bone mineral density obtained from the 3D-SHAPER® were adjusted by a post-processing algorithm to take into account an underestimation in femoral strength that can occur when converting the 2D images into 3D models. After post-processing of the 3D-SHAPER output, the 3D femur model was divided into mesh elements to create a detailed representation of the femur structure. Each element of the mesh is a 10-node tetrahedron with the target size for each element being 3 mm, ensuring a detailed and accurate model. The material card used in this model is mesh-sensitive and requires an average element size of 3 mm, as validated in our previous study [29]. The modeling pipeline, which was automated using Python open-source packages (v3.9), utilized a commercial software (Ansa 22.0.1; Beta CAE Systems, Root, Switzerland) for meshing. The FEMs were material mapped as described by Enns-Bray et al., utilizing a nonlinear material model that incorporates strain rate sensitivity and accounts for tension–compression asymmetry [29]. Briefly, the material properties of bone were mapped to the mesh using the bone density data from the scans. To do this, bone density was calibrated to ash density which was then subsequently converted to apparent density, and this was then used to calculate Young’s modulus. Three-dimensional interpolation was applied following the correction of partial volume artifacts using the material mapping method B [30]. The material mapping steps to obtain Young’s modulus assigned to the elements are described by the equations summarized in Fig. 1A [31] [32] [33]. The conversion equation from apparent density to Young’s modulus was initially developed based on trabecular bone experiments but has since been validated for the whole range of bone densities in subject-specific FEMs [30, 34, 35]. Our previous study, which applied this conversion equation to compare FEM predictions with experimental data on whole-bone stiffness and local strain, found a slope close to unity and an offset near zero, with both differences being statistically insignificant [30]. Additionally, femoral strength estimates derived from FEMs using this density–modulus relationship have shown superior performance over aBMD in distinguishing hip fracture risk in prior in vivo studies [11, 17].

In our previous study involving 4621 subjects, the sideways fall configuration with a loading of –5° for the adduction angle and 0° for internal rotation, as shown in Fig. 1B, demonstrated greater discriminatory power for predicting hip fracture risk than aBMD [17]. The distal part of the bone was the center of the condyles of a mean femur mesh [36], determined by overlaying the subject’s femur onto an average femur. It was then hinged to allow rotation solely around an axis perpendicular to the femoral shaft. Two rigid supports were used to hold the femur in place during the simulation. The supports at the greater trochanter and femoral head were modelled as rigid bodies and offset with frictionless contact from the surface of the femur. The frictionless contact is to ensure that the supports do not resist or interfere with the bone’s movement, ensuring accurate simulation. All degrees of freedom were constrained at the greater trochanter support. The femoral head support was allowed to move only in a downward direction with a boundary prescribed motion of 1 m/s as observed in experimental and computational fall models [37, 38]. The FEMs were processed in a commercially available explicit finite element solver (LS-Dyna, R12.2.1, ANSYS, Inc, Canonsburg, PA, USA). Femoral strength was recorded as the peak resultant force at the femoral head during the simulation, as shown in Fig. 1C. Force–time curves from all simulations were visually inspected to check for potential FEM modeling errors. The peak force for each simulation was extracted using an open-source Python package, *lasso-python* (v 1.5.2).

### Statistical analysis

Baseline characteristics, including age, weight, height, BMI, and *T*-score, were compared across ethnicities (Chinese, Malay, and Indian) separately for males and females. Descriptive statistics, including means and standard deviations (SD), were calculated for each variable. A one-way analysis of variance (ANOVA) was conducted using the *scipy* Python package (v1.7.1). The *p*-value obtained from the ANOVA test indicates whether there are statistically significant differences in the means for continuous variables between different ethnicities. aBMD, *T*-score, and femoral strength were adjusted for age. Statistical significance was set at a *p*-value of less than 0.05. Locally weighted scatterplot smoothing (LOWESS) [39], obtained using the *statsmodels* Python package (v0.12.2), was used to determine the trend between age and aBMD, FRAX-HFP, TSTT, and femoral strength. Additionally, ANCOVA was performed to assess the statistical significance of these LOWESS trends with respect to the covariate, “Age.” A distribution of femoral strength based on FRAX-HFP was analyzed, categorized by gender and ethnicity, to explore whether the stratification using these biomarkers overlaps or has a potential synergistic effect. The gender and ethnic relationships of FRAX-HFP and femoral strength were analyzed with respect to *T*-scores. These distributions were assessed in relation to different thresholds: the osteoporotic *T*-score threshold of − 2.5, the osteopenic threshold of − 1, the 2% threshold for FRAX-HFP [40], and the femoral strength thresholds of 3 kN for females and 3.5 kN for males, based on a Korean population [41].

## Results

**Fig. 2:**
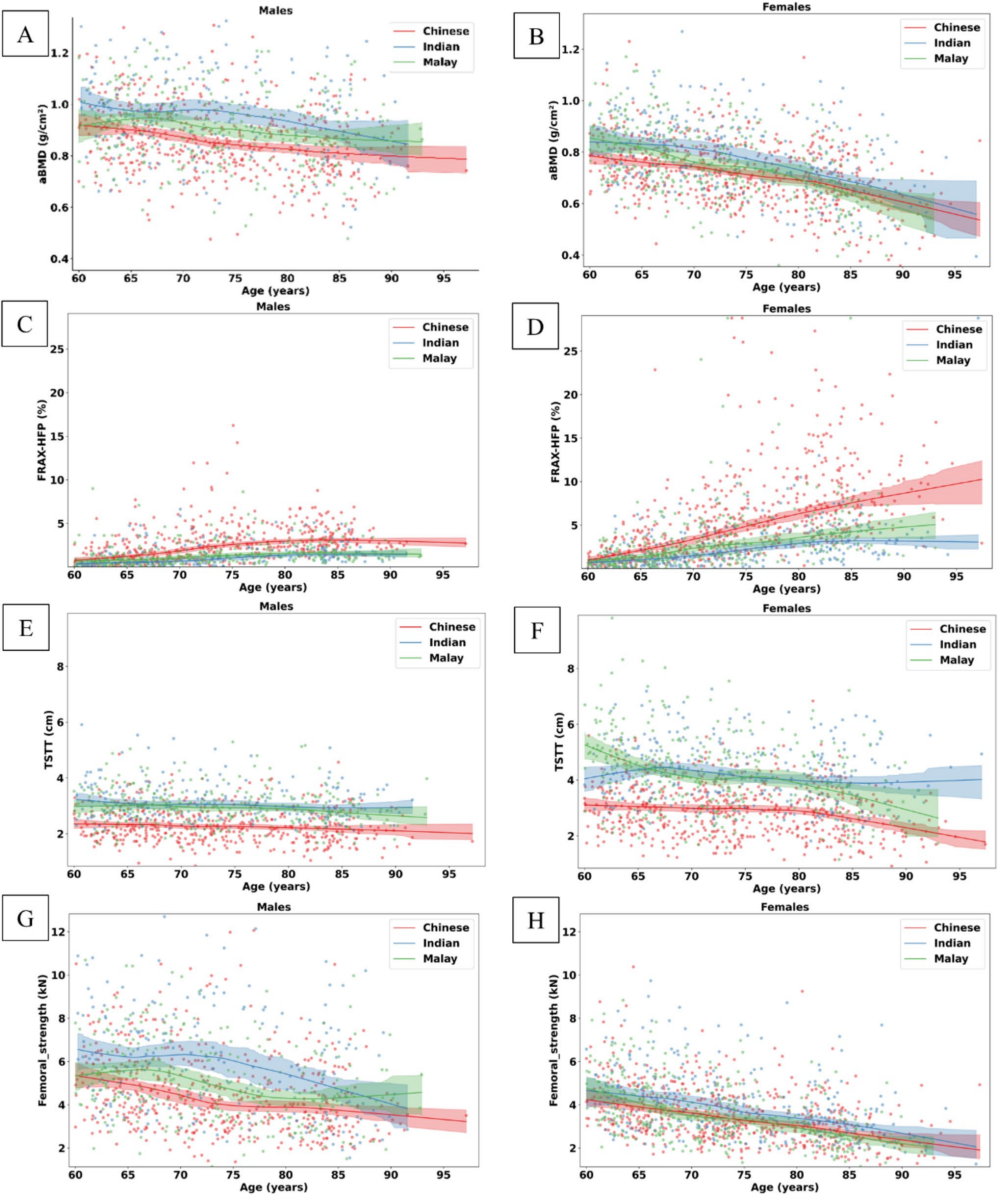
Gender and ethnic specific age-related trajectories in **A**, **B**; aBMD, **C**, **D**; FRAX-HFP score, **E**, **F**; TSTT, **G**, **H**; femoral strength, for males and females, respectively. The shaded regions indicate the 95% confidence interval for a given trajectory line. aBMD, areal bone mineral density; FRAX-HFP, fracture risk assessment tool probability of hip fracture over a 10-year period; TSTT, trochanteric soft tissue thickness

A total of 31 subjects were excluded due to motion artifacts in the DXA scans (*n* = 18) and FEM modeling errors (*n* = 13), resulting in the analysis of 2204 subjects (1224 females and 980 males). Table 1 summarizes the demographic information, along with aBMD, FRAX-HFP, TSTT, and femoral strength for the subjects. Age differences between males of the three ethnicities were not statistically significant (*p* = 0.6123), while this difference was significant between females (*p* < 0.0001). Differences in weight, height, BMI, TSTT, and FRAX-HFP were statistically significant across ethnicities for both genders (all *p* < 0.01). After adjusting for age, aBMD, *T*-score, and femoral strength continued to show significant differences between the three ethnic groups for both genders (all *p* < 0.0001) (Table 1). The trajectory lines for aBMD, TSTT, and femoral strength were comparatively lower for the Chinese population, while the trajectory lines for FRAX-HFP were higher for Chinese subjects compared to Malays and Indians (Fig. 2) (all *p*-values < 0.0001).

**Table 1:** Descriptive statistics for the cohort in the study

Variable	Pooled	Chinese	Indian	Malay	*p*-value
Number of males	980	494	213	273	
Age (years)	73.45 ± 8.34	73.70 ± 8.14	73.32 ± 8.82	73.10 ± 8.31	0.6123
Weight (kg)	67.98 ± 12.58	65.29 ± 10.65	71.17 ± 14.33	70.37 ± 13.30	< 0.0001
Height (cm)	164.80 ± 6.75	164.66 ± 6.74	166.01 ± 7.06	164.12 ± 6.41	0.0073
BMI (kg/m^2^)	25.00 ± 4.24	24.09 ± 3.87	25.71 ± 4.32	26.08 ± 4.47	< 0.0001
TSTT (cm)	2.68 ± 0.74	2.31 ± 0.60	3.13 ± 0.69	2.99 ± 0.67	< 0.0001
FRAX-HFP (%)	2.00 ± 1.81	2.56 ± 2.05	1.34 ± 1.27	1.52 ± 1.34	< 0.0001
aBMD (g/cm^2^)*	0.89 ± 0.14	0.86 ± 0.13	0.95 ± 0.15	0.91 ± 0.14	< 0.0001
*T*-score*	− 1.32 ± 1.10	− 1.57 ± 1.00	− 0.88 ± 1.20	− 1.22 ± 1.08	< 0.0001
Femoral strength (kN)*	4.99 ± 1.97	4.56 ± 1.76	5.89 ± 2.28	5.05 ± 1.82	< 0.0001
Number of females	1224	594	305	325	
Age (years)	73.71 ± 8.37	74.20 ± 8.30	74.67 ± 8.61	71.91 ± 8.01	< 0.0001
Weight (kg)	59.21 ± 12.79	55.08 ± 9.50	62.74 ± 12.84	63.46 ± 15.39	< 0.0001
Height (cm)	151.74 ± 6.40	152.84 ± 6.06	152.07 ± 6.70	149.41 ± 6.12	< 0.0001
BMI (kg/m^2^)	25.71 ± 5.32	23.57 ± 3.83	27.07 ± 4.98	28.35 ± 6.31	< 0.0001
TSTT (cm)	3.61 ± 1.27	2.95 ± 0.92	4.21 ± 1.06	4.25 ± 1.37	< 0.0001
FRAX-HFP (%)	4.23 ± 4.50	5.64 ± 5.03	2.66 ± 2.67	3.14 ± 4.01	< 0.0001
aBMD (g/cm^2^)*	0.75 ± 0.12	0.72 ± 0.11	0.78 ± 0.13	0.75 ± 0.13	< 0.0001
*T*-score*	− 1.46 ± 1.08	− 1.64 ± 0.99	− 1.13 ± 1.11	− 1.43 ± 1.12	< 0.0001
Femoral strength (kN)*	3.67 ± 1.25	3.54 ± 1.13	4.06 ± 1.39	3.55 ± 1.24	< 0.0001

*BMI* body mass index, *aBMD* areal bone mineral density at Total hip, *FRAX-HFP* fracture risk assessment tool probability of hip fracture over a 10-year period; *TSTT* trochanteric soft tissue thickness

*Adjusted for age

**Fig. 3:**
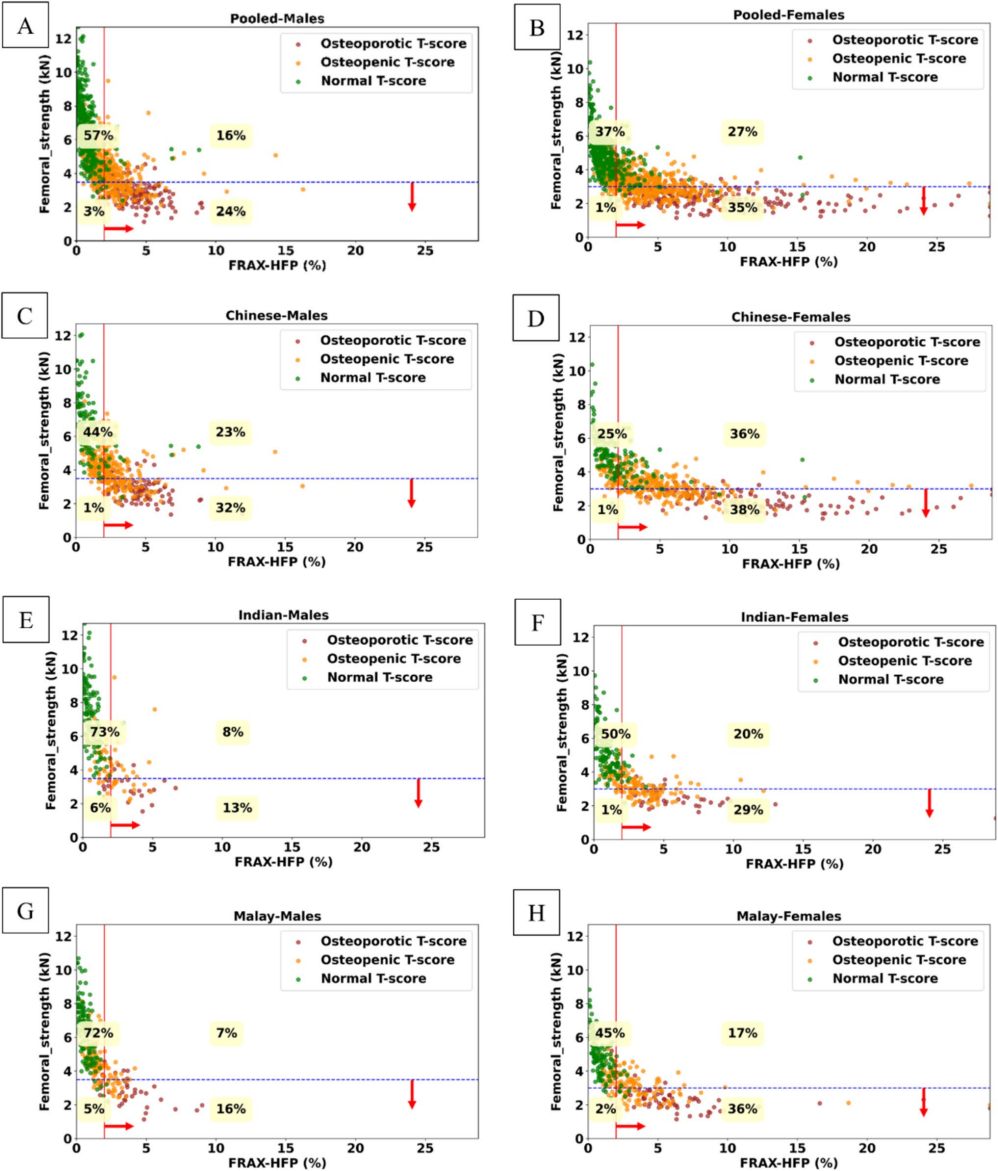
Gender and ethnic specific distribution of femoral strength as a function of FRAX-HFP score. **A**, **B,** Pooled; **C**, **D**, Chinese; **E**, **F**, Indian; **G**, **H**, Malay; for males and females, respectively. Solid red line (vertical): FRAX-HFP threshold at 2% [40]. Dashed blue line (horizontal): fragile bone threshold (females, 3 kN; males, 3.5 kN) [41]. The red arrows represent at-risk region based on the respective thresholds

**Fig. 4:**
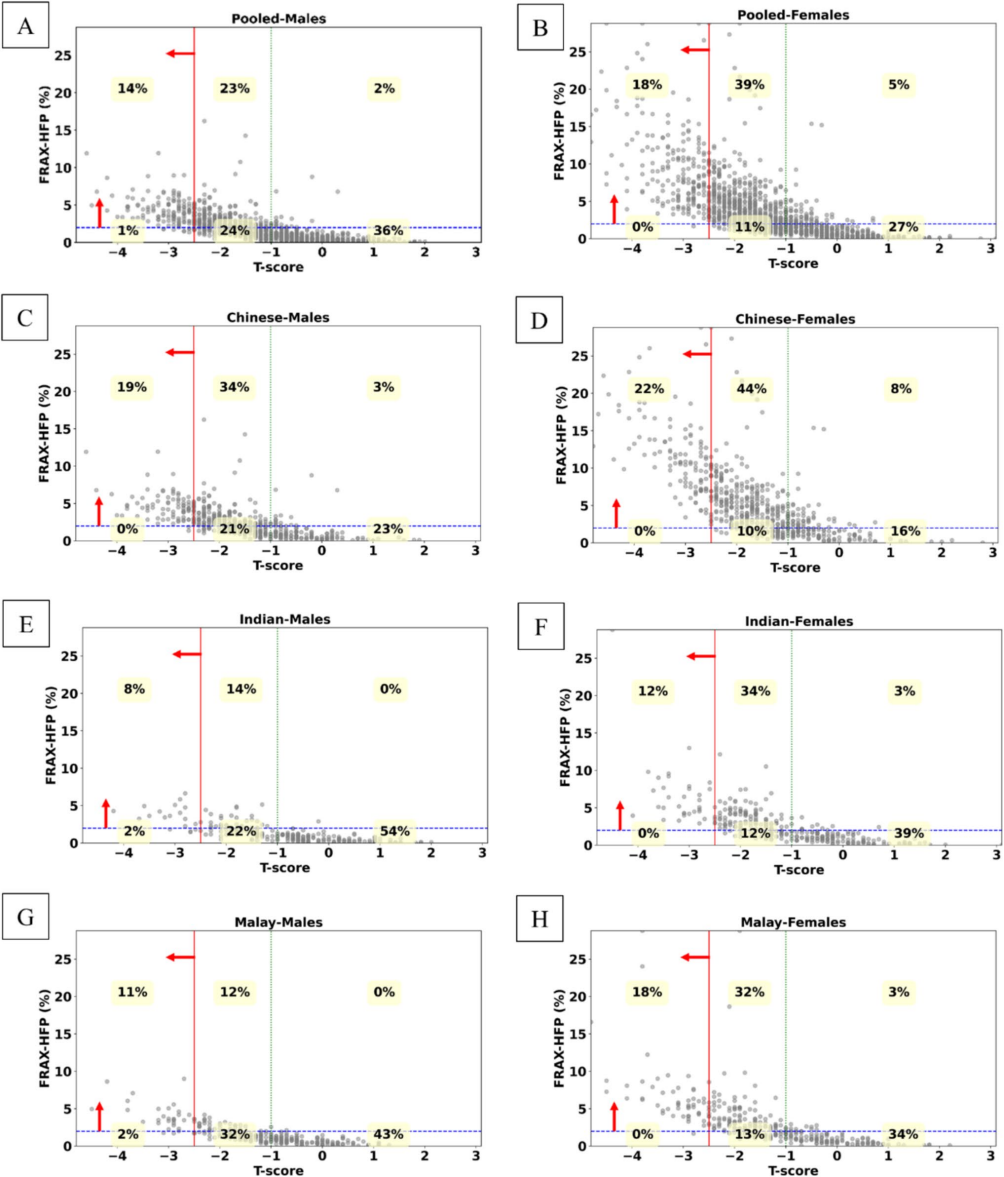
Gender and ethnic specific distribution of FRAX-HFP with respect to *T*-score. **A**, **B**, Pooled; **C**, **D**, Chinese; **E**, **F**, Indian; **G**, **H,** Malay; for males and females, respectively. Solid red line (vertical): osteoporotic *T*-score threshold (*T*-score = − 2.5). Dashed green line (vertical): normal *T*-score threshold (*T*-score = − 1). Dashed blue line (horizontal): FRAX-HFP threshold at 2% [40]. The red arrows represent at-risk region based on the respective threshold

**Fig. 5:**
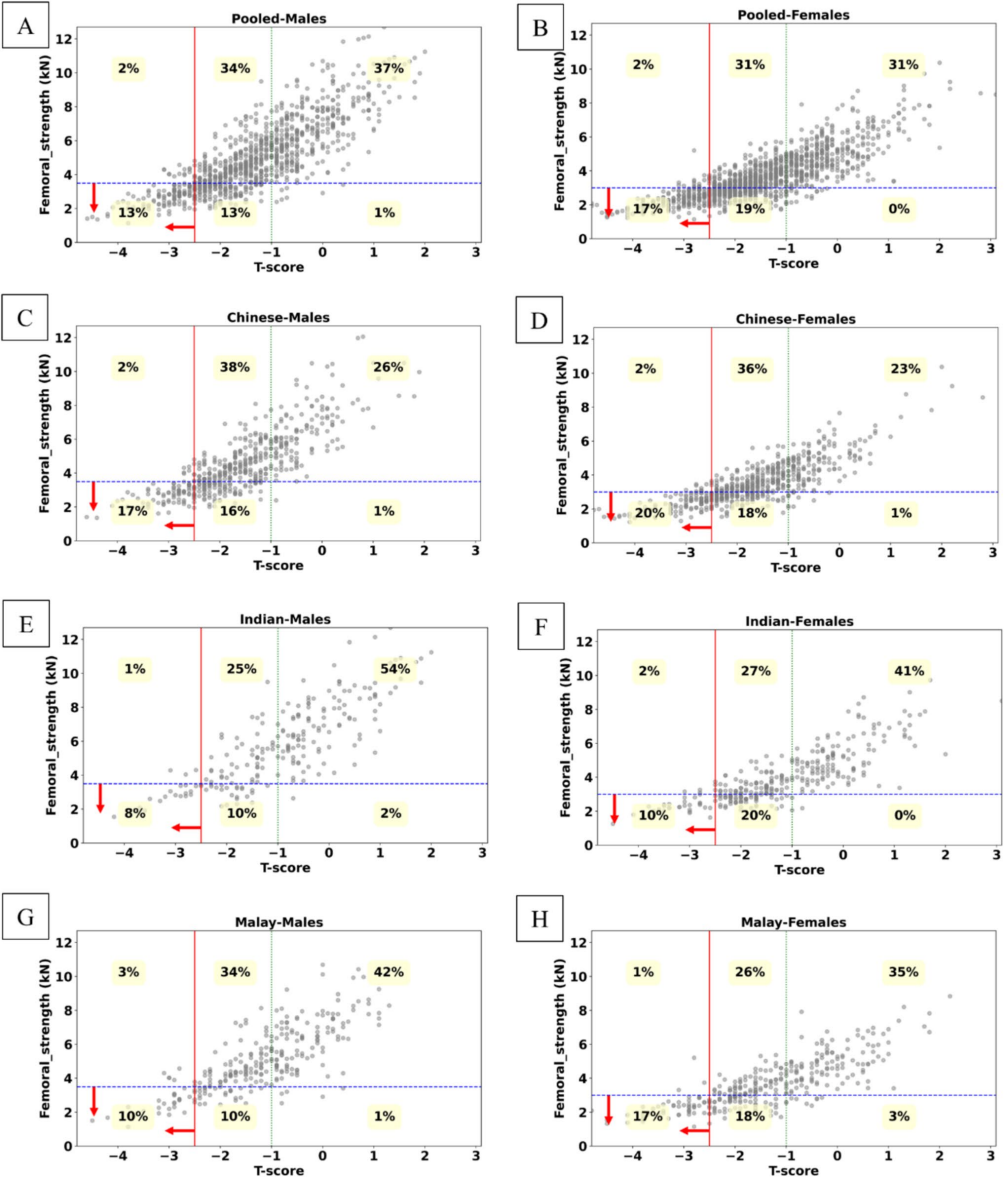
Gender and ethnic specific distribution of femoral strength with respect to *T*-score. **A**, **B**, Pooled; **C**, **D**, Chinese; **E**, **F**, Indian; **G**, **H**, Malay; for males and females, respectively. Solid red line (vertical): osteoporotic *T*-score threshold (*T*-score = − 2.5). Dashed green line (vertical): normal *T*-score threshold (*T*-score = − 1). Dashed blue line (horizontal): fragile bone threshold (females, 3 kN; males, 3.5 kN) [41]. The red arrows represent at risk region based on the respective threshold

We compared the classification of hip fracture risk between FRAX-HFP and femoral strength (Fig. 3), FRAX-HFP and *T*-score (Fig. 4), and femoral strength and *T*-score (Fig. 5) by gender and ethnicity. In both the pooled male and pooled female groups, FRAX-HFP and femoral strength identified different subjects above the selected thresholds [40, 41], with FRAX-HFP flagging a higher percentage of females (62%) compared to males (40%), while femoral strength identified 36% of females and 27% of males as above the thresholds. The *T*-score threshold identified fewer subjects at risk, with 15% in males and 18% in females. Additionally, FRAX-HFP identified 25% more males and 44% more females at risk compared to *T*-score, while femoral strength flagged an additional 14% of males and 19% of females. Across all methods, Chinese subjects were consistently identified as the highest risk group, followed by Malays and Indians, regardless of gender.

## Discussion

The primary aim of this study was to investigate the age-related trajectories of aBMD, FRAX-HFP scores, TSTT, and femoral strength in a diverse, multi-ethnic (Chinese, Indian, Malay) cohort of community-dwelling older adults in Singapore. Additionally, we examined the relationship between femoral strength and FRAX-HFP with *T*-score thresholds—the latter being the current clinical standard for a densitometric diagnosis of osteoporosis. We found that the age-related trajectories of aBMD, FRAX-HFP, TSTT, and femoral strength differed by gender and ethnicity. Specifically, compared to Malays and Indians, Chinese individuals showed higher risk metrics, such as lower aBMD, higher FRAX-HFP, thinner TSTT, and lower femoral strength. With respect to the secondary aim, the study showed that osteopenic or normal *T*-score participants were among the at-risk individuals identified by both biomarkers and that FRAX-HFP and femoral strength do not always identify the same individuals at risk, i.e., some individuals flagged by one measure (e.g., high FRAX-HFP) may not be flagged by the other. Combining these measures may provide a more nuanced approach to fracture risk estimation.

We found that females exhibited lower aBMD, higher FRAX-HFP, thinner TSTT, and lower femoral strength, all of which are associated with an increased risk of hip fractures. Furthermore, the trajectory lines for FRAX-HFP and TSTT for both genders clearly separated Chinese from other ethnic groups studied. Chinese ethnicity has lower aBMD, higher FRAX-HFP, thinner TSTT, and lower femoral strength in both genders compared to Indians and Malays. These results are in line with population data showing that Chinese females had a higher age-adjusted hip fracture rate than Indian and Malays in the Singaporean population [42]. These findings emphasize the importance of taking gender and ethnicity into account when determining hip fracture risk.

*T*-score thresholds have been observed to have lower sensitivity in capturing hip fracture risk [4–6, 17]. Both FRAX-HFP (which considers 12 clinical risk factors) and femoral strength (which is shown to have better discriminatory power than aBMD [11, 16, 17, 20]) identified individuals at risk in the osteopenic and normal *T*-score range in our study. The analysis also indicated that FRAX-HFP and femoral strength identified different individuals at risk for the specified thresholds. For the subset of individuals identified as at risk, FRAX-HFP identified a higher number of individuals compared to femoral strength for the Chinese ethnicity, while this was reversed in the case of Indian and Malays. The higher number of Chinese individuals identified by FRAX-HFP may be attributed to the demographic factors included in the FRAX-HFP score calculations. Our findings also suggest that femoral strength captures the biomechanical properties of the femur, which contributes to fracture risk independently of gender and ethnicity. Both FRAX-HFP scores and femoral strength reflected the overall population trend, with the Chinese being identified at higher risk than Indians and Malays. These results should be further validated with actual fracture outcomes to better understand the specified thresholds and their significance across different genders and ethnic groups.

This study has several limitations. First, the cohort lacks data on incident fractures, which could have been useful in validating the findings. Additionally, the Korean femoral strength thresholds [41] were applied to a multi-ethnic Singaporean population investigated in this study, but since these thresholds have not been validated with incident fracture data, further validation is needed to assess their discriminatory capacity. Despite these limitations, this study is the first to investigate age-related trends in bone health risk factors within a diverse Singaporean population, comparing FRAX-HFP scores with FEM-derived femoral strength. Future studies incorporating incident fracture data are necessary to confirm these findings.

## Conclusion

This study has several clinical and public health-related implications. It reinforces that *T*-scores, while valuable, are insufficient as a standalone measure for identifying individuals at high risk of hip fractures. Incorporating femoral strength and FRAX-HFP scores into clinical workflows could enhance risk stratification. Femoral strength, derived from DXA-based finite element models, provides biomechanical insights that are independent of clinical risk factors. The observed age-related trends are consistent with hip fracture rates in the Singaporean population. The pronounced vulnerability of Chinese participants to fractures calls for tailored interventions and preventive strategies, including targeted screening and public health initiatives to address modifiable risk factors. For Indians and Malays, lower FRAX-HFP scores and stronger femurs indicate a potentially lower fracture burden, yet individual risk assessments remain essential. The findings also highlight the need for sex-specific thresholds and interventions. Special attention should be given to women, particularly those who are postmenopausal in fracture prevention programs, given their higher FRAX-HFP scores and lower femoral strength. Validation through long-term epidemiological data and incident fracture outcomes is necessary to confirm these findings.

## References

[1] Ensrud KE (2013) Epidemiology of fracture risk with advancing age. J Gerontol A Biol Sci Med Sci 68:1236–1242. 10.1093/gerona/glt092 23833201 10.1093/gerona/glt092

[2] Aarden JJ, van der Esch M, Engelbert RHH et al (2017) Hip fractures in older patients: trajectories of disability after surgery. J Nutr Health Aging 21:837–842. 10.1007/s12603-016-0830-y28717815 10.1007/s12603-016-0830-y

[3] Haentjens P, Magaziner J, Colón-Emeric CS et al (2010) Meta-analysis: excess mortality after hip fracture among older women and men. Ann Intern Med 152:380–390. 10.1059/0003-4819-152-6-201003160-00008 20231569 10.1059/0003-4819-152-6-201003160-00008 PMC3010729

[4] Schuit SCE, van der Klift M, Weel AEAM et al (2004) Fracture incidence and association with bone mineral density in elderly men and women: the Rotterdam study. Bone 34:195–202. 10.1016/j.bone.2003.10.001 14751578 10.1016/j.bone.2003.10.001

[5] Wainwright SA, Marshall LM, Ensrud KE et al (2005) Hip fracture in women without osteoporosis. J Clin Endocrinol Metab 90:2787–2793. 10.1210/jc.2004-1568 15728213 10.1210/jc.2004-1568

[6] Stone KL, Seeley DG, Lui L-Y et al (2003) BMD at multiple sites and risk of fracture of multiple types: long-term results from the study of osteoporotic fractures. J Bone Miner Res 18:1947–1954. 10.1359/jbmr.2003.18.11.1947 14606506 10.1359/jbmr.2003.18.11.1947

[7] Marques A, Ferreira RJO, Santos E et al (2015) The accuracy of osteoporotic fracture risk prediction tools: a systematic review and meta-analysis. Ann Rheum Dis 74:1958–1967. 10.1136/annrheumdis-2015-207907 26248637 10.1136/annrheumdis-2015-207907

[8] Jha D, Chandran M, Hong N et al (2024) Discriminatory accuracy of fracture risk assessment tool in Asian populations: a systematic review and meta-analysis. J Bone Metab 31:296–315. 10.11005/jbm.24.781 39701109 10.11005/jbm.24.781 PMC11658842

[9] Hernandez CJ, Keaveny TM (2006) A biomechanical perspective on bone quality. Bone 39:1173–1181. 10.1016/j.bone.2006.06.001 16876493 10.1016/j.bone.2006.06.001 PMC1876764

[10] Adams AL, Fischer H, Kopperdahl DL et al (2018) Osteoporosis and hip fracture risk from routine computed tomography scans: the fracture, osteoporosis, and CT utilization study (FOCUS): osteoporosis and hip fracture risk from routine CT scans. J Bone Miner Res 33:1291–1301. 10.1002/jbmr.3423 29665068 10.1002/jbmr.3423 PMC6155990

[11] Fleps I, Pálsson H, Baker A et al (2022) Finite element derived femoral strength is a better predictor of hip fracture risk than aBMD in the AGES Reykjavik study cohort. Bone 154:116219. 10.1016/j.bone.2021.116219 34571206 10.1016/j.bone.2021.116219

[12] Keyak JH, Sigurdsson S, Karlsdottir G et al (2011) Male–female differences in the association between incident hip fracture and proximal femoral strength: a finite element analysis study. Bone 48:1239–1245. 10.1016/j.bone.2011.03.682 21419886 10.1016/j.bone.2011.03.682 PMC3095704

[13] Kopperdahl DL, Aspelund T, Hoffmann PF et al (2014) Assessment of incident spine and hip fractures in women and men using finite element analysis of CT scans: incident fracture assessment using FEA OF CT scans. J Bone Miner Res 29:570–580. 10.1002/jbmr.2069 23956027 10.1002/jbmr.2069 PMC3925753

[14] Michalski AS, Besler BA, Burt LA, Boyd SK (2021) Opportunistic CT screening predicts individuals at risk of major osteoporotic fracture. Osteoporos Int 32:1639–1649. 10.1007/s00198-021-05863-0 33566138 10.1007/s00198-021-05863-0

[15] Orwoll ES, Marshall LM, Nielson CM et al (2009) Finite element analysis of the proximal femur and hip fracture risk in older men. J Bone Miner Res 24:475–483. 10.1359/jbmr.081201 19049327 10.1359/JBMR.081201 PMC2659519

[16] Yosibash Z, Trabelsi N, Buchnik I et al (2023) Hip fracture risk assessment in elderly and diabetic patients: combining autonomous finite element analysis and machine learning. J Bone Miner Res 38:876–886. 10.1002/jbmr.4805 36970838 10.1002/jbmr.4805

[17] Praveen AD, Jha D, Baker A et al (2025) Comparison of the time-dependent discriminatory accuracy of femoral strength and bone mineral density for predicting future hip and major osteoporotic fractures: a 16-year follow-up of the AGES-Reykjavik cohort. Osteoporos Int. 10.1007/s00198-025-07503-3 40353869 10.1007/s00198-025-07503-3 PMC12208951

[18] Engelke K, Adams JE, Armbrecht G et al (2008) Clinical use of quantitative computed tomography and peripheral quantitative computed tomography in the management of osteoporosis in adults: the 2007 ISCD official positions. J Clin Densitom 11:123–162. 10.1016/j.jocd.2007.12.010 18442757 10.1016/j.jocd.2007.12.010

[19] Whitmarsh T, Humbert L, De Craene M et al (2011) Reconstructing the 3d shape and bone mineral density distribution of the proximal femur from dual-energy x-ray absorptiometry. IEEE Trans Med Imaging 30:2101–2114. 10.1109/TMI.2011.2163074 21803681 10.1109/TMI.2011.2163074

[20] Grassi L, Väänänen SP, Ristinmaa M et al (2017) Prediction of femoral strength using 3d finite element models reconstructed from DXA images: validation against experiments. Biomech Model Mechanobiol 16:989–1000. 10.1007/s10237-016-0866-2 28004226 10.1007/s10237-016-0866-2 PMC5422489

[21] Grassi L, Väänänen SP, Jehpsson L et al (2023) 3D finite element models reconstructed from 2D dual-energy X-ray absorptiometry (DXA) images improve hip fracture prediction compared to areal BMD in osteoporotic fractures in men (MrOS) Sweden cohort. J Bone Miner Res 38:1258–1267. 10.1002/jbmr.4878 37417707 10.1002/jbmr.4878

[22] Grassi L, Väänänen SP, Voss A et al (2025) DXA-based 3D finite element models predict hip fractures better than areal BMD in elderly women. Bone 195:117457. 10.1016/j.bone.2025.117457 40086683 10.1016/j.bone.2025.117457

[23] Humbert L, Martelli Y, Fonolla R et al (2017) 3D-DXA: assessing the femoral shape, the trabecular macrostructure and the cortex in 3D from DXA images. IEEE Trans Med Imaging 36:27–39. 10.1109/TMI.2016.2593346 27448343 10.1109/TMI.2016.2593346

[24] Dudle A, Gugler Y, Pretterklieber M et al (2023) 2D–3D reconstruction of the proximal femur from DXA scans: evaluation of the 3D-shaper software. Front Bioeng Biotechnol 11:1111020. 10.3389/fbioe.2023.1111020 36937766 10.3389/fbioe.2023.1111020 PMC10014626

[25] Qasim M, López Picazo M, Ruiz Wills C et al (2024) 3D-DXA based finite element modelling for femur strength prediction: evaluation against QCT. J Clin Densitom 27:101471. 10.1016/j.jocd.2024.101471 38306806 10.1016/j.jocd.2024.101471

[26] Cheung C-L, Ang SB, Chadha M et al (2018) An updated hip fracture projection in Asia: the Asian federation of osteoporosis societies study. Osteoporos Sarcopenia 4:16–21. 10.1016/j.afos.2018.03.003 30775536 10.1016/j.afos.2018.03.003 PMC6362950

[27] Sing C-W, Lin T-C, Bartholomew S et al (2023) Global epidemiology of hip fractures: secular trends in incidence rate, post-fracture treatment, and all-cause mortality. J Bone Miner Res 38:1064–1075. 10.1002/jbmr.4821 37118993 10.1002/jbmr.4821

[28] Gupta P, Man REK, Fenwick EK et al (2020) Rationale and methodology of the population health and eye disease profile in elderly Singaporeans study [PIONEER]. Aging Dis 11:1444–1458. 10.14336/AD.2020.0206 33269099 10.14336/AD.2020.0206 PMC7673841

[29] Enns-Bray WS, Bahaloo H, Fleps I et al (2018) Material mapping strategy to improve the predicted response of the proximal femur to a sideways fall impact. J Mech Behav Biomed Mater 78:196–205. 10.1016/j.jmbbm.2017.10.033 29172124 10.1016/j.jmbbm.2017.10.033

[30] Helgason B, Gilchrist S, Ariza O et al (2016) The influence of the modulus–density relationship and the material mapping method on the simulated mechanical response of the proximal femur in side-ways fall loading configuration. Med Eng Phys 38:679–689. 10.1016/j.medengphy.2016.03.006 27185044 10.1016/j.medengphy.2016.03.006

[31] Keyak JH, Lee IY, Skinner HB (1994) Correlations between orthogonal mechanical properties and density of trabecular bone: use of different densitometric measures. J Biomed Mater Res 28:1329–1336. 10.1002/jbm.820281111 7829563 10.1002/jbm.820281111

[32] Schileo E, Dall’Ara E, Taddei F et al (2008) An accurate estimation of bone density improves the accuracy of subject-specific finite element models. J Biomech 41:2483–2491. 10.1016/j.jbiomech.2008.05.017 18606417 10.1016/j.jbiomech.2008.05.017

[33] Morgan EF, Bayraktar HH, Keaveny TM (2003) Trabecular bone modulus–density relationships depend on anatomic site. J Biomech 36:897–904. 10.1016/S0021-9290(03)00071-X 12757797 10.1016/s0021-9290(03)00071-x

[34] Schileo E, Taddei F, Malandrino A et al (2007) Subject-specific finite element models can accurately predict strain levels in long bones. J Biomech 40:2982–2989. 10.1016/j.jbiomech.2007.02.010 17434172 10.1016/j.jbiomech.2007.02.010

[35] Grassi L, Schileo E, Taddei F et al (2012) Accuracy of finite element predictions in sideways load configurations for the proximal human femur. J Biomech 45:394–399. 10.1016/j.jbiomech.2011.10.019 22079387 10.1016/j.jbiomech.2011.10.019

[36] Zhang J, Besier TF (2017) Accuracy of femur reconstruction from sparse geometric data using a statistical shape model. Comput Methods Biomech Biomed Eng 20:566–576. 10.1080/10255842.2016.1263301 10.1080/10255842.2016.1263301 27998170

[37] Fleps I, Fung A, Guy P et al (2019) Subject-specific ex vivo simulations for hip fracture risk assessment in sideways falls. Bone 125:36–45. 10.1016/j.bone.2019.05.004 31071479 10.1016/j.bone.2019.05.004

[38] Fleps I, Guy P, Ferguson SJ et al (2019) Explicit finite element models accurately predict subject-specific and velocity-dependent kinetics of sideways fall impact. J Bone Miner Res 34:1837–1850. 10.1002/jbmr.3804 31163090 10.1002/jbmr.3804

[39] Cleveland WS (1979) Robust locally weighted regression and smoothing scatterplots. J Am Stat Assoc. 10.2307/2286407

[40] Chandran M, Ganesan G, Tan KB et al (2021) Using health-economic evidence to support policy-level decision-making in Singapore—sensitivity analysis that provides further confidence in fracture probability-based cost-effective intervention thresholds. Osteoporos Int 32:787–789. 10.1007/s00198-021-05876-9 33566137 10.1007/s00198-021-05876-9

[41] Hong N, Lee DC, Khosla S et al (2020) Comparison of vertebral and femoral strength between White and Asian adults using finite element analysis of computed tomography scans. J Bone Miner Res 35:2345–2354. 10.1002/jbmr.4149 32750185 10.1002/jbmr.4149 PMC9260814

[42] Yong EL, Ganesan G, Kramer MS et al (2019) Hip fractures in Singapore: ethnic differences and temporal trends in the new millennium. Osteoporos Int 30:879–886. 10.1007/s00198-019-04839-5 30671610 10.1007/s00198-019-04839-5

